# Host Genetic Factors Associated with Vaginal Microbiome Composition in Kenyan Women

**DOI:** 10.1128/mSystems.00502-20

**Published:** 2020-07-28

**Authors:** Supriya D. Mehta, Drew R. Nannini, Fredrick Otieno, Stefan J. Green, Walter Agingu, Alan Landay, Yinan Zheng, Lifang Hou

**Affiliations:** a Division of Epidemiology & Biostatistics, University of Illinois at Chicago School of Public Health, Chicago, Illinois, USA; b Center for Global Oncology, Institute of Global Health, Northwestern University Feinberg School of Medicine, Chicago, Illinois, USA; c Department of Preventive Medicine, Northwestern University Feinberg School of Medicine, Chicago, Illinois, USA; d Nyanza Reproductive Health Society, Kisumu, Kenya; e Genome Research Core, University of Illinois at Chicago, College of Medicine, Chicago, Illinois, USA; f Department of Internal Medicine, Rush University College of Medicine, Chicago, Illinois, USA; University of North Carolina at Charlotte

**Keywords:** vaginal microbiome, vaginal microbiota, bacterial vaginosis, *Gardnerella vaginalis*, *Lactobacillus crispatus*, *Lactobacillus iners*, *L. crispatus*, *L. iners*, *G. vaginalis*, Shannon diversity index, community state type, genome wide association study, pathway analysis, Toll-like receptors, innate immune response, Kenya, community state type, GWAS

## Abstract

Bacterial vaginosis (BV) affects 20% of women worldwide and is associated with adverse reproductive health outcomes and increased risk for HIV. Typically, BV represents a shift in the vaginal microbiome from one that is dominated by *Lactobacillus* to one that is diverse. Persistent racial differences in BV and diverse vaginal microbiome composition overlap with racial disparities in risks for HIV and sexually transmitted infection, especially among women of African descent. Risk factors for BV and nonoptimal vaginal microbiome include sexual practices, yet racial differences persist when adjusted for behavioral factors, suggesting a host genetic component. Here, we perform a genome-wide association study on vaginal microbiome traits in Kenyan women. Linear regression and logistic regression were performed, adjusting for age and principal components of genetic ancestry, to evaluate the association between Lactobacillus crispatus, Lactobacillus iners, Gardnerella vaginalis, Shannon diversity index, and community state type (CST) with host genetic single nucleotide polymorphisms (SNPs). We identified novel genomic loci associated with the vaginal microbiome traits, though no SNP reached genome-wide significance. During pathway enrichment analysis, Toll-like receptors (TLRs), cytokine production, and other components of innate immune response were associated with L. crispatus, L. iners, and CST. Multiple previously reported genomic loci were replicated, including *IL-8* (Shannon, CST), *TIRAP* (*L. iners*, Shannon), *TLR2* (Shannon, CST), *MBL2* (*L. iners*, G. vaginalis, CST), and *MYD88* (*L. iners*, Shannon). These genetic associations suggest a role for the innate immune system and cell signaling in vaginal microbiome composition and susceptibility to nonoptimal vaginal microbiome.

**IMPORTANCE** Globally, bacterial vaginosis (BV) is a common condition in women. BV is associated with poorer reproductive health outcomes and HIV risk. Typically, BV represents a shift in the vaginal microbiome from one that is dominated by *Lactobacillus* to one that is diverse. Despite many women having similar exposures, the prevalence of BV and nonoptimal vaginal microbiome is increased for women of African descent, suggesting a possible role for host genetics. We conducted a genome-wide association study of important vaginal microbiome traits in Kenyan women. We identified novel genetic loci and biological pathways related to mucosal immunity, cell signaling, and infection that were associated with vaginal microbiome traits; we replicated previously reported loci associated with mucosal immune response. These results provide insight into potential host genetic influences on vaginal microbiome composition and can guide larger longitudinal studies, with genetic and functional comparison across microbiome sites within individuals and across populations.

## Abstract (precis)

Globally, bacterial vaginosis (BV) is a common condition in women. BV is associated with poorer reproductive health outcomes and HIV risk. Typically, BV represents a shift in the vaginal microbiome from one that is dominated by *Lactobacillus* to one that is diverse. Despite many women having similar exposures, the prevalence of BV and nonoptimal vaginal microbiome is increased for women of African descent, suggesting a possible role for host genetics. We conducted a genome-wide association study of important vaginal microbiome traits in Kenyan women. We identified novel genetic loci and biological pathways related to mucosal immunity, cell signaling, and infection that were associated with vaginal microbiome traits; we replicated previously reported loci associated with mucosal immune response. These results provide insight into potential host genetic influences on vaginal microbiome composition and can guide larger longitudinal studies, with genetic and functional comparison across microbiome sites within individuals and across populations.

## INTRODUCTION

Bacterial vaginosis (BV) is a condition of clinical and public health significance. BV is prevalent in 20 to 50% of women in sub-Saharan Africa (1). In a meta-analysis, Atashili et al. estimated the relative risk of HIV acquisition to be 1.6 times higher for women with BV, equating to a population attributable fraction of 15% due to the high prevalence in the population (2). BV is also associated with an increased likelihood of sexually transmitted infections (STIs) and adverse pregnancy outcomes (3, 4). BV represents a polymicrobial shift in the vaginal microbiome, often from a community dominated by *Lactobacillus* to one that is diverse, with multiple species of anaerobic bacteria (5). In particular, Lactobacillus crispatus enrichment has been shown to be protective against BV, HIV, and STIs (6).

BV is considered a sexually enhanced infection, and individuals with new or multiple sex partners, those who engage in unprotected vaginal sex, or those whose sex partner is uncircumcised are at an increased risk of BV (3, 7–9). In addition to sexual risk factors, nonsexual risk factors that are associated with BV include intravaginal and vaginal hygiene practices (10), cigarette smoking (11), and race/ethnicity (12). In numerous studies, women of African descent have increased risk of BV and nonoptimal vaginal microbiome composition (13). These associations with race persist even when controlling for sociodemographics and sexual practices (14, 15), leading to the hypothesis that genetic factors may influence the vaginal microbiome composition and subsequently BV (16).

To date, a limited number of studies have examined the host genetic contribution to the vaginal flora and BV. Primarily these studies have focused on host differences in candidate genes responsible for inflammatory mucosal immune response in other infectious conditions (16, 17). As reviewed by Turovskiy et al., studies have targeted single nucleotide polymorphisms (SNPs) in (i) Toll-like receptor (TLR) genes because of their role in recognizing potential threats and initiating inflammatory responses and activating other immune cells and (ii) proinflammatory and inflammatory cytokines (e.g., interleukin 1β [IL-1β], tumor necrosis factor alpha [TNF-α]) (17). Most of these studies, however, evaluated SNPs in relation to BV or to specific taxa associated with BV (e.g., Gardnerella vaginalis, Atopobium vaginae), and none of the studies measured the vaginal microflora or host genetic contribution comprehensively, such as with cultivation-independent molecular microbial characterization or a genome-wide association study (GWAS), respectively.

To address this gap, we conducted GWAS on vaginal microbiome traits among native Kenyan women. We hypothesized that by conducting GWAS, we would identify novel genomic loci associated with vaginal microbiome traits, furthering our understanding of the underlying genetic factors. Identifying genetic variants may aid in elucidating the biological mechanisms associated with these complex vaginal microbiome traits and associated diseases. Complementary to GWAS, we conducted replication analyses of SNPs previously reported to be associated with vaginal microflora or BV.

## RESULTS

### Study sample.

Among 171 women included in this analysis, the prevalence of BV at baseline was 22% (Table 1). More than half (53%) of the women were herpes simplex virus 2 (HSV-2) seropositive, and 10% were HIV positive, which is in keeping with the prevalence of HIV among women in this age range in the general population in this region of western Kenya (18). Lactobacillus crispatus was present in 25% of samples (28% mean relative abundance), Lactobacillus iners was present in 83% of samples (mean relative abundance 45%), and Gardnerella vaginalis was present in 75% of samples (25% mean relative abundance).

**TABLE 1:** Baseline participant characteristics and vaginal microbiome composition overall and stratified by community state type^*a*^

Characteristic	No. of participants (%) with characteristic or specified value			
	Total (*n* = 171)	CST I (*n* = 14)	CST-III (*n* = 77)	CST-IV^*b*^ (*n* = 77)
Median age in yrs (IQR)	22 (20–25)	24.5 (19–28)	23 (20–25)	22 (20–25)
HIV status				
Negative	152 (89.9)	14 (100)	70 (92.1)	65 (85.5)
Positive	17 (10.1)		6 (7.9)	11 (14.5)
Missing	2		1	1
HSV-2 status				
Negative	81 (47.4)	12 (85.7)	39 (50.6)	28 (36.4)
Positive	90 (52.6)	2 (14.3)	38 (49.4)	49 (63.6)
Circumcised male sex partner	91 (53.2)	6 (42.9)	43 (55.8)	40 (52.0)
BV (Nugent score of 7 to 10) at baseline	37 (21.6)	0 (0)	4 (5.2)	33 (42.9)
Proportion of visits with BV over follow-up				
0%	89 (52.1)	14 (100)	49 (63.6)	25 (32.5)
25%	22 (12.9)		10 (13.0)	11 (14.3)
25%−50%	33 (20.2)		11 (14.3)	21 (27.3)
50%−75%	19 (11.1)		7 (9.1)	12 (15.5)
100%	8 (4.7)			8 (10.4)
Presence of L. crispatus at baseline	42 (24.6)	14 (100)	22 (28.6)	6 (7.8)
Mean RA of L. crispatus if present (SD)	28.2 (35.3)	72.5 (21.6)	7.71 (13.0)	0.21 (0.34)
Presence of *L. iners* at baseline	142 (83.0)	5 (35.7)	77 (100)	58 (75.3)
Mean RA of *L. iners* if present (SD)	44.8 (42.4)	1.84 (3.24)	80.3 (23.2)	2.99 (5.11)
Presence of *G. vaginalis* at baseline	129 (75.4)	7 (50.0)	46 (59.7)	73 (94.8)
Mean RA of *G. vaginalis* if present (SD)	23.1 (24.8)	20.9 (15.0)	6.81 (10.8)	33.7 (26.7)
Median Shannon diversity index (IQR)	0.94 (0.30–1.82)	0.80 (0.33–0.98)	0.34 (0.16–0.83)	1.84 (1.20–2.17)

a CST, community state type; IQR, interquartile range; BV, bacterial vaginosis; RA, relative abundance; SD, standard deviation.

b CST-IV combined subtypes A (*n* = 9), B (*n* = 62), and C (*n* = 6). Statistics stratified by CST are not reported for three observations (two for CST-II and one for CST-V).

### Genome-wide association results.

The genomic control (GC) inflation factor, λ, values were 1.01, 1.00, 1.00, 1.00, and 1.00 for L. crispatus, L. iners, G. vaginalis, the Shannon diversity index, and CST, respectively, after adjusting for age and the first three principal components. Upon visual inspection of the quantile-quantile (Q-Q) plots (see Fig. S1 in the supplemental material), the observed *P* values do not deviate from the null, except at the extreme tail. Together with the genomic inflation factors, the Q-Q plots indicate proper control of population stratification.

10.1128/mSystems.00502-20.1 FIG S1 (A to E) Q-Q plots for five vaginal microbiome traits measured in 171 Kenyan women. Inspection of Q-Q plots for each microbiome trait evaluated indicate proper control of population stratification. Download FIG S1, TIF file, 0.2 MB. Copyright © 2020 Mehta et al. 2020 Mehta et al. This content is distributed under the terms of the Creative Commons Attribution 4.0 International license.

**FIG 1:**
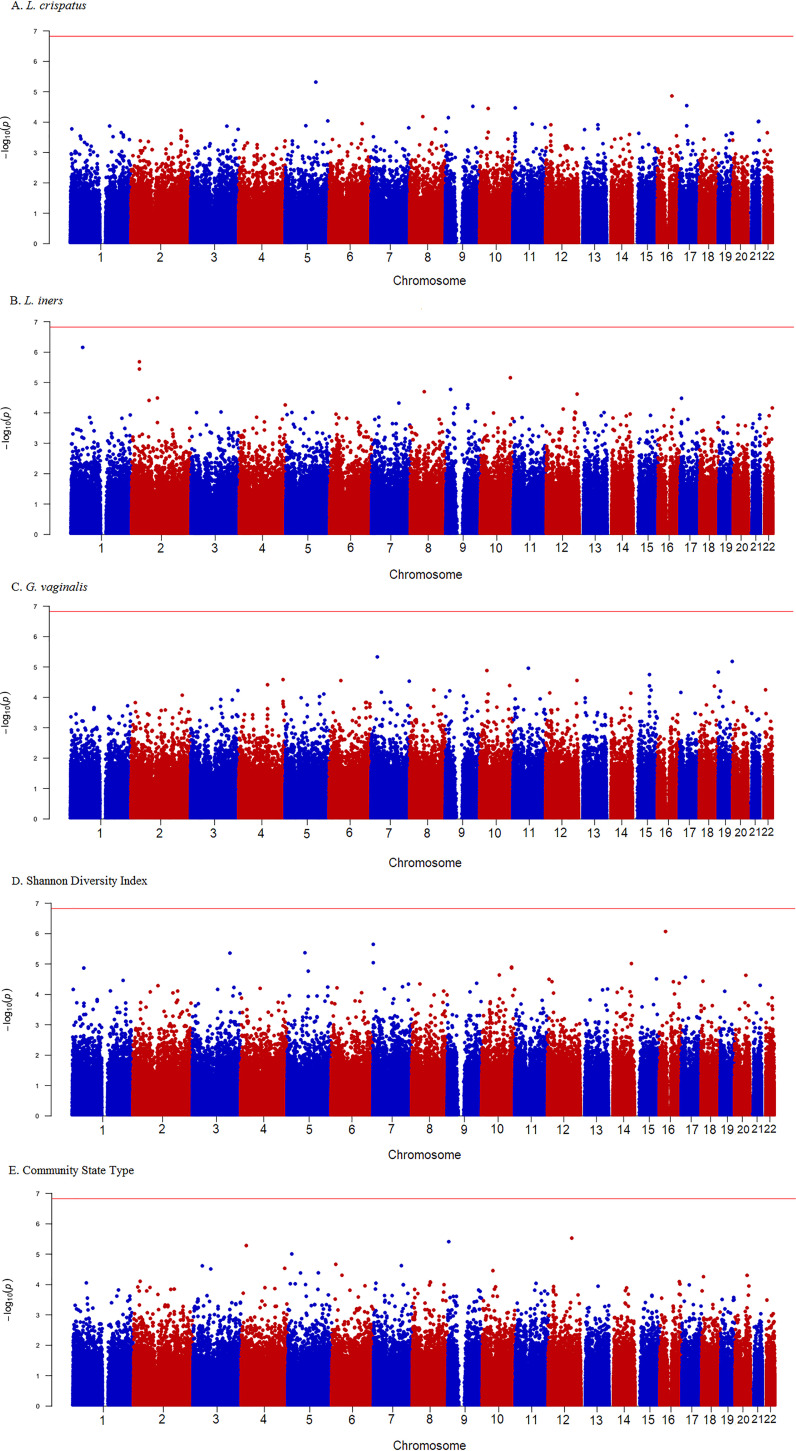
(A to E) Manhattan plots for single nucleotide polymorphisms associated with vaginal microbiome traits. The *x* axis corresponds to the genomic position, and the *y* axis shows the −log_10_ of the *P* value. The horizontal red line corresponds to the genome-wide significance threshold line corresponds to the genome-wide significance threshold.

Figure 1A to E display the Manhattan plots of the genome-wide *P* values for each vaginal microbiome trait. Table 2 summarizes the top SNPs (*P* < 1 × 10^−5^) for each vaginal microbiome trait and minor allele frequencies (MAF) derived from our data. Overall, no SNP reached genome-wide significance (0.05/336,151 = 1.49E−07). The SNP with the lowest adjusted *P* value associated with the presence of L. crispatus was rs73330467 (*P* = 4.79 × 10^−6^; Fig. 1A), located in an intergenic region between *LOC101927488* and *GRAMD2B* on chromosome 5. The minor allele G (MAF = 0.07) was associated with an 11.6-fold increased odds of detecting L. crispatus. The most significant SNP associated with relative abundance of *L. iners* was rs527430 (*P* = 6.98 × 10^−7^; Fig. 1B) on chromosome 1, located in an intergenic region between *FOXD2* and *TRABD2B*. The minor allele A (MAF = 0.06) was associated with an increase in relative abundance of *L. iners* (β = 1.05). The most significant SNP associated with *G. vaginalis* was rs1229660 (*P* = 4.65 × 10^−6^; Fig. 1C) located between *SNX10-LOC441204* on chromosome 7. The minor allele C (MAF = 0.10) was associated with a decrease in *G. vaginalis* relative abundance (β = −0.99). The most significant SNP associated with the Shannon diversity index was rs972741 (*P* = 8.52 × 10^−7^; Fig. 1D) located between *ZKSCAN2* and *HS3ST4* on chromosome 16. The minor allele A (MAF = 0.19) was associated with an increase in the Shannon diversity index (β = 0.66). The most significant SNP associated with CST was rs2302902 (*P* = 3.09 × 10^−6^; Fig. 1E) located in *ELK3* on chromosome 12. The minor allele T (MAF = 0.20) was associated with an increased likelihood of membership in lower-diversity CST-I and CST-II compared to CST-IV (β = 0.41). Sensitivity analyses were performed to further adjust for HIV and herpes simplex virus (HSV) status and yielded similar results and conclusions (see Table S1 in the supplemental material). Results for the comparison of the top SNPs between the vaginal microbiome traits are presented in Table S2. Overall, each microbiome trait was associated with at least one other top SNP, i.e., 1 for L. crispatus, 4 for *L. iners*, 4 for *G. vaginalis*, 3 for Shannon diversity index, and 5 for CST.

**TABLE 2:** Top single nucleotide polymorphisms associated with five vaginal microbiome traits

Trait and SNP^*a*^	Chr^*b*^	Position	Gene^*c*^	A1/A2^*d*^	MAF^*e*^	β	*P*^*f*^
L. crispatus							
rs73330467	5	125694420	*LOC101927488-GRAMD2B*	G/T	0.07	11.58^*g*^	4.79E−06
*L. iners*							
rs527430	1	47918821	*FOXD2-TRABD2B*	A/G	0.06	1.05	6.98E−07
rs77007265	2	31384829	*GALNT14-CAPN14*	C/A	0.08	−0.93	2.07E−06
rs17010778	2	31384974	*GALNT14-CAPN14*	C/T	0.06	−1.00	3.60E−06
rs12221275	10	122972398	*WDR11-FGFR2*	A/C	0.01	2.22	6.95E−06
*G. vaginalis*							
rs1229660	7	26437429	*SNX10-LOC441204*	C/T	0.10	−0.99	4.65E−06
rs10414170	19	57246309	*ZNF835-ZIM2-AS1*	C/A	0.30	−0.62	6.56E−06
Shannon diversity index							
rs7632135	3	154455745	*GPR149-MME*	G/A	0.24	−0.59	4.37E−06
rs3097137	5	73330562	*ARHGEF28-LINC01335*	A/G	0.20	0.56	4.25E−06
rs112627544	7	1929410	***MAD1L1***	T/G	0.42	−0.47	9.04E−06
rs6970796	7	1947895	***MAD1L1***	T/C	0.36	−0.50	2.25E−06
rs56952063	14	95107145	***SERPINA13P***	C/T	0.08	−0.90	9.65E−06
rs972741	16	25468083	*ZKSCAN2-HS3ST4*	A/G	0.19	0.66	8.52E−07
Community state type							
rs419816	5	52571758	*LOC257396-FST*	C/T	0.13	0.45	9.99E−06
rs1929353	9	3759975	*RFX3-AS1-GLIS3*	G/A	0.24	0.35	9.51E−06
rs2302902	12	96617304	***ELK3***	T/C	0.20	0.41	3.09E−06

a L. crispatus is modeled as presence/absence; *L. iners* and *G. vaginalis* are modeled as continuous and quantiles of inverse log-transformed relative abundance, respectively. SNP, single nucleotide polymorphism.

b Chr, chromosome.

c Gene name is in boldface if the SNP is located within the gene.

d A1/A2, allele 1/allele 2.

e MAF, minor allele frequency.

f SNPs with a *P* value of <1 × 10^−5^ for each trait are included in the table.

g Reported as odds ratio.

10.1128/mSystems.00502-20.3 TABLE S1 Sensitivity analysis of the top GWAS SNPs adjusting for HIV and HSV-2 status. SNP, single nucleotide polymorphism; Chr, chromosome; A1/A2, allele 1/allele 2. *^a^*Reported as odds ratio. Download Table S1, DOCX file, 0.01 MB. Copyright © 2020 Mehta et al. 2020 Mehta et al. This content is distributed under the terms of the Creative Commons Attribution 4.0 International license.

10.1128/mSystems.00502-20.4 TABLE S2 Comparison of top SNPs across vaginal microbiome traits. *P* values corresponding to the top SNPs identified during single trait analysis are shown in italics. SNPs with *P* < 3.13 × 10^−3^ (0.05/16) are shown in boldface. Shannon, Shannon diversity index; CST, community state type. Download Table S2, DOCX file, 0.02 MB. Copyright © 2020 Mehta et al. 2020 Mehta et al. This content is distributed under the terms of the Creative Commons Attribution 4.0 International license.

### Results from imputed SNPs.

**FIG 2:**
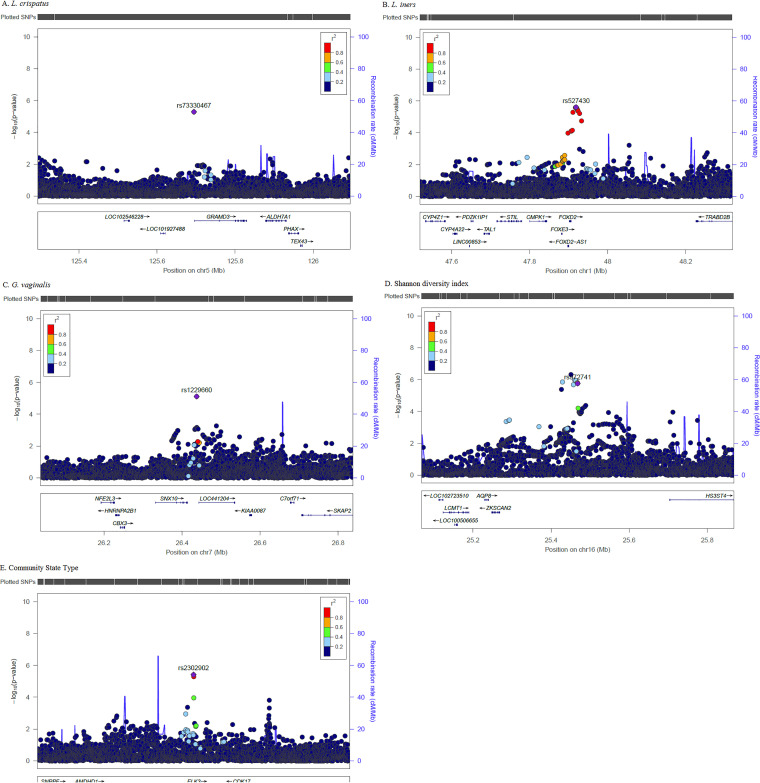
(A to E) Regional association plots of the top genomic loci associated with vaginal microbiome traits. *P* values (−log_10_) of the GWAS (solid circles) on the *y* axis are plotted against the genomic positions of each SNP on the *x* axis for each microbiome trait. Genes in the region are shown below. The linkage disequilibrium (LD) values (*r*^2^) between the lead SNP and the other SNPs are indicated in different colors.

We performed genotype imputation in order to expand genomic coverage and interrogate additional SNPs not directly genotyped. After removing SNPs with low imputation quality and low frequency (R-squared [Rsq] < 0.30 and MAF < 1%), several imputed SNPs (but no additional genomic regions) were identified. Figure 2 presents the regional SNP association plots for each of the vaginal microbiome traits. Within the *ZKSCAN2-HS3ST4* intergenic region on chromosome 16, several imputed SNPs were more significantly associated with the Shannon diversity index than those directly genotyped (Fig. 2E). The most significant SNP in this region was rs115869045 (*P* = 4.77 × 10^−7^; Rsq = 0.70).

### Conditional analyses.

We conducted conditional analyses for the genomic loci in which the most significant SNP was identified for each vaginal microbiome trait to determine whether additional SNPs (±400 kb) contribute to the observed associations. Figure S2 displays the regional SNP associations conditioning on the most significant directly genotyped SNP by including the SNP as a covariate in the regression model. As shown in each plot, all of the immediate SNP associations reduced toward the null, suggesting that the SNPs identified are the leading SNPs for the observed associations.

10.1128/mSystems.00502-20.2 FIG S2 (A to E) Regional conditional association plots of the top genomic loci associated with vaginal microbiome traits. *P* values (−log_10_) of the GWAS (solid circles) on the *y* axis are plotted against the genomic positions of each SNP on the *x* axis for each microbiome trait. Genes in the region are shown below. The linkage disequilibrium (LD) values (*r*^2^) between the lead SNP and the other SNPs are indicated in different colors. Download FIG S2, TIF file, 0.9 MB. Copyright © 2020 Mehta et al. 2020 Mehta et al. This content is distributed under the terms of the Creative Commons Attribution 4.0 International license.

### Analysis of previously reported loci.

**FIG 3:**
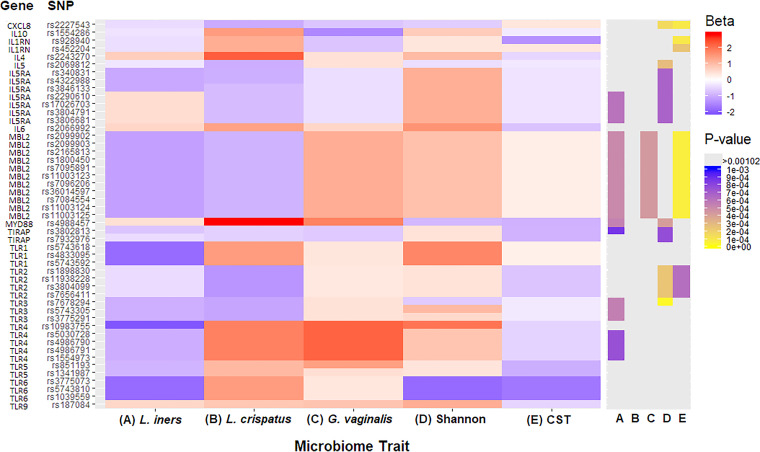
Heatmap summarizing beta coefficients for the most significant SNPs within 100 kbp of previously reported SNPs. The heatmap on the left represents the direction and magnitude of the coefficients (beta) for the SNPs within 100 kb associated with five vaginal microbiome traits: (A) relative abundance of *L. iners*, (B) presence of L. crispatus, (C) relative abundance of *G. vaginalis*, (D) Shannon diversity index, (E) community state type (CST), with CST-IV as the reference. Negative coefficients are shaded in blue, and positive coefficients are shaded in red, with deeper intensity representing the magnitude of the coefficient. The heatmap on the right represents the *P* value of the corresponding coefficients for SNPs and vaginal microbiome traits (A to E). All associations shown have *P* values of <0.05. The Bonferroni cutoff for significance is <0.00102, and these *P* values are shaded yellow to purple. *P* values of >0.00102 to 0.05 are shaded gray.

We investigated the associations of previously reported SNPs in relation to BV or vaginal microflora to determine whether these associations are relevant in a Kenyan population (19–26). Figure 3 and Table S3 summarize these findings. Overall, 49 autosomal SNPs were identified from previous studies, of which 45 SNPs exhibited high imputation quality (Rsq ≥ 0.30). When analyzed, 7 SNPs for *L. iners*, 8 SNPs for L. crispatus, 6 SNPs for *G. vaginalis*, 10 SNPs for the Shannon diversity index, and 8 SNPs for CST had a *P* < 0.05 in our sample. These findings, however, were not significant after applying a Bonferroni correction (0.05/49 = 0.00102).

10.1128/mSystems.00502-20.5 TABLE S3 Analysis of previously reported loci. Note that this is an Excel file. Download Table S3, XLSX file, 0.03 MB. Copyright © 2020 Mehta et al. 2020 Mehta et al. This content is distributed under the terms of the Creative Commons Attribution 4.0 International license.

To replicate genomic loci associated with the microbiome traits, we extracted the most significant imputed SNP within 100 kb of the previously reported SNPs for each microbiome trait in our study. This approach resulted in 6 genomic regions for *L. iners*, 1 genomic region for *G. vaginalis*, 7 genomic regions for Shannon diversity index, and 4 genomic regions for CST that were significant after Bonferroni correction (Fig. 3 and Table S3). No regions were significant after multiple testing correction for L. crispatus. The replicated genomic regions include *IL1RN* (CST), *IL-5* (Shannon), *IL5RA* (*L. iners*, Shannon), *IL-8* (Shannon, CST), *TIRAP* (*L. iners*, Shannon), *TLR2* (Shannon, CST), *TLR3* (*L. iners*, *Shannon*), *TLR4* (*L. iners*), *MBL2* (*L. iners*, *G. vaginalis*, CST), and *MYD88* (*L. iners*, Shannon).

### Gene, pathway, and disease/phenotype analyses.

During gene-based analyses, no genes remained significant after multiple testing correction for any of the microbiome traits (Table S4). Table 3 presents the top five Kyoto Encyclopedia of Genes and Genomes (KEGG) and Reactome pathways from WebGestalt for each vaginal microbiome trait. Four pathways were significantly associated with L. crispatus after Benjamini-Hochberg multiple testing adjustment, including G-protein-coupled receptor (GPCR) ligand binding and neutrophil degranulation. Five pathways were associated with *L. iners*: 4 related to TLR cascades, and one to the TLR upstream component (MyD88:Mal TIRAP [MyD88-adaptor-like TIR {Toll-interleukin 1 receptor} domain containing adaptor protein]). Two biological pathways related to GPCR signaling were associated with *G. vaginalis*, as well as “neutrophil degranulation,” though it did not meet Bonferroni significance. Similarly, two pathways were associated with CST, including the most significant pathway “Class B/2 (secretin family receptors),” and one pathway was associated with the Shannon diversity index, “Class B/2 (secretin family receptors).”

**TABLE 3:** Results of pathway analysis: top KEGG and Reactome pathways associated with vaginal microbiome traits

Trait and pathway^*a*^	Observed no. of genes/total no. of genes	*P* value	Adjusted *P* value^*b*^
L. crispatus			
Metabolism of amino acids and derivatives	29/370	7.27E−06	1.10E−02
GPCR ligand binding	32/457	2.46E−05	1.37E−02
PCP/CE pathway	11/92	1.50E−04	3.97E−02
Beta-catenin-independent WNT signaling	14/145	2.04E−04	4.72E−02
Neutrophil degranulation	30/479	3.12E−04	5.31E−02
*L. iners*			
Toll-like receptor cascades	17/155	8.41E−06	9.76E−03
MyD88:MAL(TIRAP) cascade initiated on plasma membrane	13/95	8.82E−06	9.76E−03
Toll-like receptor TLR6:TLR2 cascade	13/95	8.82E−06	9.76E−03
Toll-like receptor TLR1:TLR2 cascade	13/98	1.25E−05	9.76E−03
Toll-like receptor 2 (TLR2) cascade	13/98	1.25E−05	9.76E−03
*G. vaginalis*			
Signaling by GPCR	66/1162	5.35E−07	1.46E−03
GPCR downstream signaling	63/1100	7.45E−07	1.46E−03
Neutrophil degranulation	29/479	3.11E−04	2.44E−01
G alpha (s) signaling events	31/536	4.29E−04	2.80E−01
Formation of the cornified envelope	12/129	5.23E−04	2.93E−01
Shannon diversity index			
Class B/2 (secretin family receptors)	12/95	1.89E−05	3.71E−02
RNA polymerase II transcription	62/1292	6.53E−05	5.12E−02
Gene expression (transcription)	66/1430	1.19E−04	6.52E−02
Neuronal system	24/368	1.99E−04	6.52E−02
Cleavage of growing transcript in the termination region	8/67	6.80E−04	9.52E−02
Community state type			
Human T-cell leukemia virus 1 infection	20/255	8.77E−05	9.30E−03
Class B/2 (secretin family receptors)	11/95	1.23E−04	1.06E−02
GPCR ligand binding	29/457	1.25E−04	1.06E−02
Posttranslational modification: synthesis of GPI-anchored proteins	10/92	4.17E−04	1.89E−02
Transmission across chemical synapses	17/227	4.95E−04	2.12E−02

a PCP/CE, planar cell polarity/convergent extension; GPI, glycosylphosphatidylinositol.

b *P* values are Benjamini-Hochberg adjusted.

10.1128/mSystems.00502-20.6 TABLE S4 Top genes from gene level analysis for each vaginal microbiome trait. Bonferroni corrected *P* value, 2.36 × 10^−6^ (0.05/21,213). Download Table S4, DOCX file, 0.01 MB. Copyright © 2020 Mehta et al. 2020 Mehta et al. This content is distributed under the terms of the Creative Commons Attribution 4.0 International license.

Table 4 summarizes the top five DisGeNET, GLAD4U, Online Mendelian Inheritance in Man (OMIM), and Human Phenotype Ontology terms from WebGestalt for each vaginal microbiome trait. Five phenotypes were significantly associated with L. crispatus after Benjamini-Hochberg multiple testing adjustment, including “Bacterial infections” (*P* = 0.011) as the most significant phenotype. Three phenotypes were associated with *L. iners*, with “Autosomal dominant inheritance” the most significant (*P* = 0.018). No phenotypes were significantly associated with *G. vaginalis*. One phenotype was associated with the Shannon diversity index, “Abnormality of the female genitalia” (*P* = 0.037). Five phenotypes were associated with CST, including “Abnormality of the integument” (*P* = 0.00023).

**TABLE 4:** Results of disease/phenotype analysis: top phenotypes associated with vaginal microbiome traits

Trait and phenotype	Observed no. of genes/total no. of genes	*P* value	Adjusted *P* value^*a*^
L. crispatus			
Bacterial infections	23/260	9.25E−06	1.10E−02
Meckel syndrome (MKS)	17/164	1.78E−05	1.37E−02
Hypertension, pulmonary	15/137	2.88E−05	1.37E−02
Varicose veins	11/79	3.64E−05	1.45E−02
Gram-positive bacterial infections	17/181	6.29E−05	2.15E−02
*L. iners*			
Autosomal dominant inheritance	72/1403	3.17E−05	1.78E−02
Growth abnormality	88/1822	3.89E−05	1.91E−02
Abnormality of the cardiovascular system	86/1796	6.49E−05	2.83E−02
Aplasia/hypoplasia involving the vertebral column	8/55	3.08E−04	8.05E−02
Abnormality of hair density	20/267	3.35E−04	8.05E−02
*G. vaginalis*			
Pancreatic neoplasm	11/88	6.51E−05	8.52E−02
Carcinoma, pancreatic ductal	13/139	2.95E−04	2.44E−01
Pancreatic neoplasms	19/281	9.08E−04	3.56E−01
Neoplasm of the endocrine system	9/89	1.42E−03	4.11E−01
Thyroid neoplasms	13/165	1.48E−03	4.11E−01
Shannon diversity index			
Abnormality of the female genitalia	33/505	1.31E−05	3.71E−02
Abnormality of female internal genitalia	29/444	4.38E−05	5.12E−02
Abnormal internal genitalia	30/473	5.57E−05	5.12E−02
Abnormality of the uterus	15/176	1.92E−04	6.52E−02
Abnormality of the ovary	16/196	1.96E−04	6.52E−02
Community state type			
Serotonin syndrome	23/134	1.18E−11	4.39E−08
Abnormality of the integument	99/1920	5.16E−08	1.01E−04
Autosomal dominant inheritance	74/1403	1.33E−06	1.50E−03
Prader-Willi syndrome	20/195	1.70E−06	1.50E−03
Abnormality of the skin	80/1572	1.92E−06	1.50E−03

a *P* values are Benjamini-Hochberg adjusted.

## DISCUSSION

We observed associations between genetic and biological pathways in native Kenyan women with biological relevance to host immunity, cell signaling, and infection, supporting the role of host genetics in interindividual variability in vaginal microbiome traits.

In GWAS, no SNP reached genome-wide significance. The most significant SNP associated with CST (rs2302902) is located in an intron of *ELK3*, a member of the ETS transcription factor family, which has not been previously associated with any vaginal microbiome traits or BV. Proteins in the *ELK3* subfamily are recruited by serum response factor and participate in transcription regulation, and they have been associated with various cancers (27–29), including human papillomavirus (HPV)-positive tumors of oropharyngeal cancer (30) and HPV16 in cervical tumors (31). In meta-analysis, molecular and clinical measures of BV are associated with a twofold increase in risk of high-grade cervical epithelial neoplasia (CIN) or cancer (32). Nonoptimal vaginal microbiome leads to mucosal disruption and a proinflammatory environment that can facilitate HPV acquisition (33), but there may also be underlying shared or synergistic mechanism with ELK3. Other SNPs identified in GWAS occurred primarily in intergenic regions that have not previously been associated with BV or vaginal microbiome traits, and they should be assessed for replication in future studies.

BV was absent from CST-I (L. crispatus dominant) and infrequent (5.1%) in CST-III. Although CST-IV accounted for 89% of BV cases in this sample, 57% of women in CST-IV did not have BV. This is in keeping with many other studies: women with diverse CSTs are much more likely to have BV, but as many as 50% of women with these CSTs do not have BV (34). Host genetics may partially explain this variability. For example, women with similar vaginal bacterial colonization (such as those within CST-IV) may have different response to bacterial colonization depending on genetic traits. For women who mount an inflammatory response, this immune response may potentiate BV rather being solely a response to BV. In our study, restricting analyses to HIV uninfected women did not change our findings. This is not entirely unexpected, as associations between HIV and subsequent impact on the vaginal microbiome are variable, with some finding no effect (35, 36). Conversely, there are substantial data that demonstrate the temporal association between nonoptimal vaginal microbiome composition, mucosal inflammation, and subsequent HIV risk (37), and our results suggest that host genetic differences may underpin this. Well-powered longitudinal studies that examine how host genetics is associated with different CSTs, mucosal immune markers, and BV trajectories could be informative to understanding this variability in pathology within nonoptimal CSTs. Additionally, studies are needed to explore the potential long-range regulation of these SNPs on genes, as well as evaluate for expression or methylation quantitative trait locus associations.

Complex traits are polygenic, and our analysis of previously reported loci identified multiple SNPs across numerous genetic loci with Bonferroni corrected significance. Several SNPs in the *MBL2* region were significantly associated with *L. iners*, *G. vaginalis*, and CST. Kalia et al. studied these previously reported *MBL2* SNPs in association with BV in women in India due to associations between mannose binding lectin (MBL) insufficiency and increased susceptibility to infectious diseases (26). Among Brazilian women, a specific *MBL2* polymorphism was associated with increased odds of recurrent BV compared to controls (38), though a study among Italian women that screened for three specific *MBL2* SNPs found no differences for women with recurrent BV compared to controls (39). The relevance of this gene may be population specific or subject to interaction with other factors such as the prevalence of coinfections (e.g., HSV-2, HIV), sexual exposures (e.g., partner circumcision status, multiple sex partners, condom use), or nonsexual factors (e.g., cigarette smoking, intravaginal practices). Additionally, we identified SNPs in IL-1RN (CST), IL-5 (Shannon), and IL-5RA (*L. iners*, Shannon). IL-1RN (the gene for interleukin 1 receptor agonist [IL-1RA]) inhibits the inflammatory IL-1 cytokines, IL-1α and IL-1β. In a study of 62 Italian women, compared to women with Nugent scores of 0 to 3, women with BV (Nugent scores of 7 to 10) had increased IL-1RA, and increased inflammatory IL-5 was associated with decreased *Lactobacillus* (40). In candidate gene analysis, Si et al. targeted genetic variants of IL-5, finding association with increased abundances of various *Prevotella* (24). Our findings further these studies by analyzing genetic factors and vaginal microbiome traits in a novel population and by demonstrating trans-ethnic associations for these previously identified loci.

In pathway analysis, GPCR pathways were identified with L. crispatus, *G. vaginalis*, and CST, and class B/2 (secretin family receptors) within the GPCR family were identified with Shannon diversity index and CST. GPCRs play a significant role in intracellular signaling in response to a broad range of stimuli (e.g., hormones, neurotransmitters, proteins, etc.) and across a wide range of functions (e.g., growth, nutrition requirements, response to disease, etc.) (41). This likely includes vaginal microbiome modulation, and variation in GPCR signaling may be related to variations in innate immune response. LL-37 is elevated in women with BV, and in an *ex vivo* endocervical model, after application of a GPCR inhibitor, LL-37-mediated induction of IL-8 production was inhibited (42); this is relevant given that IL-8 is often elevated in women with BV (43). In analyses of previously reported SNPs, we found that SNPs in the IL-8 gene region were associated with Shannon diversity index and CST. In an experimental study, Mares et al. demonstrate that application of cervicovaginal lavage (CVL) from women with BV induced higher levels of IL-8 and NF-κB in human monocytes than CVL from women without BV (44). Among women in the CAPRISA 004 trial, those with vaginal CST-IV had increased mucosal inflammation (including elevated IL-8) and increased risk of HIV seroconversion (37). The effects of variation in GPCR signaling on innate immune response may extend to other outcomes. Among Dutch women, GWAS and pathway analysis found that genes encoding GPCR signaling were enriched for 71 Chlamydia trachomatis-seropositive women compared to 169 control women (45). GPCR signaling encompasses a broad range of actions, but in conjunction with our pathway results implicating IL-8 and TLRs and other studies showing potential roles for GPCR in cervicovaginal health, GPCR signaling merits further study as a potential pathway affecting acquisition or maintenance of vaginal L. crispatus, CST membership, and subsequent BV status.

Our pathway analysis additionally linked neutrophil degranulation with L. crispatus and *G. vaginalis*. Neutrophils are effector cells in innate immunity, and degranulation can have serious adverse consequences for the host due to release of damaging molecules. We are unaware of other studies linking neutrophil degranulation to the vaginal microbiome, BV, or other vaginal infections. However, myeloperoxidase (MPO) is a measure of neutrophils and is elevated in women with sexually transmitted infections (STIs) (46). Whether neutrophil degranulation is an important process in vaginal microbiome composition may be evaluated in subsequent studies, such as by targeting genes encoding molecules associated with this pathway (e.g., β-arrestins, Rac2) (47).

Last, pathway analysis identified “MyD88:MAL (TIRAP) cascade” with *L. iners*, and we identified SNPs within the *MyD88* gene in relation to *L. iners* and Shannon diversity index. MyD88:Mal (TIRAP) is an adaptor to MyD88, the first downstream component of TLR4 and TLR2, and part of the larger overall process for inflammatory cytokine effects (48). In pathway analysis, several TLR signaling associations were associated with *L. iners* relative abundance, and in analysis of previously reported SNPs, TLR2 and TLR3 had associations with Shannon diversity index, CST, and *L. iners*. As summarized in Table S3 in the supplemental material, several investigators have found polymorphisms in TLR genes related to altered risk of BV and enrichment of BV-related bacteria. TLRs, especially TLR2 alone or in combination with TLR1/TLR6 and TLR4, have been recognized for their association with BV and BV-related bacteria, as these TLRs precipitate release of proinflammatory cytokines and recruitment of inflammatory cells (20–23, 25). As summarized by Taylor et al., TLR gene variants can lead to inadequate or excess inflammatory immune response, which can affect disease susceptibility or progression (25). Additionally, studies are needed to evaluate the potential biological role TLRs have on vaginal microbiome composition. Given that the microbiome is a complex trait and complex traits are pleiotropic, this may provide understanding of how TLR variants contribute to risk of BV, response to BV treatment, or other outcomes of nonoptimal vaginal microbiome composition, such as adverse pregnancy outcomes.

We hypothesized that host genetic factors might be associated with vaginal microbiome traits, as has been found in the gut and airway, including associations with host genetic components encoding variants in the innate immune system (49, 50). As summarized by Thaiss et al., the innate immune system senses bacteria and their metabolic products and translates this to host physiologic responses to support organismal homeostasis (51). For example, microbiota influence the function of interleukins, potentially driving inflammation, and MyD88 expression at the epithelial barrier supports intestinal homeostasis (51). Taken together, our findings demonstrate numerous biologically relevant pathways associated with vaginal microbiome traits. However, such associations do not imply causality, and additional study is necessary to address unknown gene expression, pathways with multiple functions, and nonspecificity of gene polymorphisms or pathways with the vaginal microbiome, with larger study in multiple ethnic groups.

### Limitations.

The power of this study is limited by small sample size: in our GWAS, no SNPs reached genome-wide significance after multiple correction, and we were unable to assess genes by environment interactions. We calculated the number of associations passing a range of *P* value threshold counts (Table S5), which shows that we would not have detected significant SNPs at the genome-wide significance level (5 × 10^−8^) or the Bonferroni significance level of our study (1.49 × 10^−7^). It is highly likely we have false-negative findings due to small sample size. To understand the desired sample size for a study replicating our approach, we estimated minimum sample sizes necessary to reach the Bonferroni significance level (Table S6): GWAS study of these microbiome traits in a sample similar to ours will need a sample size of at least 188 to 376 women, and sample size varies based on trait and measure of association.

10.1128/mSystems.00502-20.7 TABLE S5 Count of top single nucleotide polymorphisms (SNPs) by *P* value threshold for each vaginal microbiome trait. *5.00 × 10^−8^ is the conventional genome-wide significance level used in genome-wide association studies. 1.49 × 10^−7^ is the Bonferroni significance level in this study. Download Table S5, DOCX file, 0.01 MB. Copyright © 2020 Mehta et al. 2020 Mehta et al. This content is distributed under the terms of the Creative Commons Attribution 4.0 International license.

10.1128/mSystems.00502-20.8 TABLE S6 Power table representing the minimum sample sizes needed to reach Bonferroni significance for each vaginal microbiome trait, expressed at three statistical power settings: 80%, 90%, and 95% power. *Bonferroni significance *P* value in our study, *P* = 1.49 × 10^−7^. **“N Extra” represents the number of samples needed in addition to the current samples in the study. SNP, single nucleotide polymorphism; Chr, chromosome. Download Table S6, DOCX file, 0.02 MB. Copyright © 2020 Mehta et al. 2020 Mehta et al. This content is distributed under the terms of the Creative Commons Attribution 4.0 International license.

We found substantial functional overlap in several biologically relevant innate immunity pathways. This may in part be driven by the compositionality of individual taxa. To mitigate this, we limited examination to a few traits of known importance in conjunction with comprehensive community characterization. We analyzed CST linearly, though multinomial or quasi-Poisson may have been more applicable given the likely unequal effects across levels; however, these approaches are not supported by genetic statistical analysis software. We also confirmed numerous SNPs in close proximity to genomic loci associated with innate immunity reported in previous studies of host genetic traits associated with BV and vaginal taxa. Although we identified novel candidate regions associated with vaginal microbiome traits, additional studies are needed to replicate our findings. Despite the limitations of small sample size, the advantage of our unbiased discovery approach is that it can more easily be combined with other future studies. Evaluation of functional impact with multiomic studies (such as gene expression, epigenetics, and fine mapping) would allow mechanistic inference and aid in elucidating regulatory elements, which could lead to translational advances in optimizing the vaginal microbiome and preventing BV and associated consequences.

### Conclusions.

Given the multiple associations of important microbiome traits with various immune signaling pathways, the composition of the vaginal microbiome (and subsequently, BV risk) may be influenced by host genetics through immune phenotypes. Replication studies and functional studies are needed to confirm and further elucidate understanding of how vaginal bacteria interact with the genetic makeup of the host to affect nonoptimal vaginal microbiome and related outcomes. Given the cooccurrence of risk for BV, nonoptimal vaginal microbiome, and HIV and STI among African and African American women, well-powered cross-national studies are needed to answer whether common variants are replicated across populations and to identify important genes by environment interactions that inform how much risk for nonoptimal vaginal microbiome may be modifiable, such as through behavioral modification or prevention or treatment of coinfections (e.g., HSV-2, HIV).

## MATERIALS AND METHODS

This study was approved by the Ethical Review Committee of Maseno University (Kisumu, Kenya; MSU/DRPC/MUERC/00054/13; 13 January 2014), and the Institutional Review Board of the University of Illinois at Chicago (USA; 2013-0511; 12 February 2014).

### Study design and participants.

Subjects in this analysis were enrolled in *Afya Jozi*, *Afya Jamii* (Kiswahili for “Healthy Pair, Healthy Community”), a prospective cohort study of heterosexual couples in Kisumu, Kenya. Recruitment and eligibility criteria have been previously published (52). Briefly, we included men aged 18 to 35 years old and their female partners aged 16 years and older, who independently confirmed a sexual relationship of at least 6 months duration. Men or women reporting antibiotic use within the past 60 days, men who were circumcised within the past 6 months, and women who reported vaginal douching within the past 7 days were temporarily excluded until sufficient time had passed. After the baseline visit, couples were scheduled for follow-up at 1 month, 6 months, and 12 months. Couples were enrolled from 1 April 1 2014 through 22 June 2016, and 12-month follow-up was completed 21 June 2017.

### Specimen collection.

At each scheduled study visit, participants underwent a standardized medical history and physical examination, followed by a personal interview to obtain sociodemographic and behavioral information. At baseline and each follow-up visit, clinicians collected vaginal swabs for assessment of BV. Gram-stained slides were evaluated according to Nugent’s criteria, and a score of 7 to 10 was defined as BV following clinical recommendations (53). Vaginal swab collection was followed by collection of CVL in 10 ml sterile water. Aliquots of 2 ml were stored at −80°C until shipment to Chicago, IL, for processing.

### Vaginal microbiome characterization.

We used the vaginal microbiome as measured from the baseline visit for this analysis, because antimicrobial treatment was provided for BV and other cervicovaginal infections which may confound subsequent vaginal microbiome observations. DNA extraction, library preparation, and sequencing were performed at the University of Illinois at Chicago Sequencing Core (UICSQC). DNA was extracted from CVL specimens using a PowerFecal DNA kit (Qiagen). A two-stage PCR protocol was used to amplify the V3-V4 variable region of bacterial 16S rRNA genes (54). Illumina MiSeq was used to sequence the amplicons after pooling, implementing V3 chemistry (600 cycles). Quality control and taxonomic annotation were conducted by University of Maryland Institute for Genomic Science (UMD IGS) following previously published protocols (55). Community state types (CSTs) were determined by the VALENCIA algorithm implemented by UMD IGS, which uses a distance-based metric to classify each sample to a CST based on the similarity of that sample to the centroid of CSTs identified in a reference set (56). Briefly, dominant taxa by CST are as follows: CST-I, L. crispatus; CST-II, Lactobacillus gasseri; CST-III, L. iners; CST-IV, diverse; CST-V, Lactobacillus jensenii (12).

### Genotyping and quality control.

Genomic DNA was obtained from 200 Kenyan women using oral swabs and was extracted using QIAamp DNA blood minikit (Qiagen). DNA extraction and isolation were performed at the Center for Population Epigenetics at Northwestern University. Study participants were genotyped using the Infinium Global Screening Array (∼640,000 markers; Illumina, Inc., San Diego, CA). The software Illumina GenomeStudio (v2.0.4; Illumina, Inc., San Diego, CA) was used to call single nucleotide polymorphisms (SNPs) at the Genomic Facility, University of Chicago. Study participants with a genotyping call rate of less than 95% were excluded from analysis as were study participants with missing vaginal microbiome traits. Due to cryptic relatedness identified during quality control, we restricted all analyses to the maximum number of unrelated individuals. Implementation of these exclusion criteria resulted in 171 study participants for analysis. SNPs with a call rate of less than 95%, a minor allele frequency of less than 1%, or a Hardy-Weinberg equilibrium *P* value of less than 10^−6^ were omitted. After these exclusion criteria, 336,151 remained for further analysis.

### Genotype imputation.

We performed genotype imputation to identify additional SNPs associated with the vaginal microbiome traits that were not directly genotyped using the Luhya in Webuye, Kenya, reference panel from the 1000 Genomes Project (57). The program Shapeit2 (58) was used to phase genotypes, and imputation was performed using Minimac3 (59). Imputed genotypes were coded as allelic dosages (estimated counts ranging from 0 to 2). Low-quality imputed SNPs, i.e., Rsq < 0.30 and MAF < 1%, were excluded from further analyses.

### Statistical analysis.

We evaluated the association between single nucleotide polymorphisms (SNPs) with five vaginal microbiome traits: (i) presence of L. crispatus, (ii) relative abundance of *L. iners*, (iii) relative abundance of *G. vaginalis*, (iv) Shannon diversity index, and (v) CST. We chose L. crispatus for its known importance in vaginal microbiome health, *L. iners* for its predominance among *Lactobacillus* species in our sample and other African samples, and *G. vaginalis* for its inclusion as a morphotype in Nugent scoring and determination of BV. For alpha diversity measures, we selected the Shannon diversity index, as all alpha diversity measures were highly correlated (Spearman correlations, 0.77 to 0.98). Prior to analyses, the relative abundance of *L. iners* and Shannon diversity index were inverse log transformed to approximate a more normal distribution (see Fig. S1 in the supplemental material). As transformation did not lead to normal approximation, the relative abundance of *G. vaginalis* was analyzed by quartile. Given the infrequency of L. crispatus (present in 24% of samples), we analyzed it as presence versus absence. Of 171 subjects in the analysis, 168 (98%) baseline vaginal microbial communities were classified as CST-I (*n* = 14; 0% with BV), CST-III (*n* = 77; 5.2% with BV), or CST-IV (*n* = 77; 43% with BV). There were three outliers (two with CST-II and one with CST-V), and these observations were excluded from CST analyses. BV was not analyzed as a separate outcome because it was colinear with CST. We analyzed CST as a linear outcome (i.e., equal distance between CST-I, CST-II, and CST-III) given the ordinal nature of BV risk and to meet parameters for statistical software.

Principal components of genetic ancestry were generated using the program EIGENSOFT (60), and the first three principal components were retained and included as covariates. Quantile-quantile (Q-Q) plots were generated for each trait to visualize test statistics, and the genomic control inflation factors were calculated. We used PLINK (v1.90) (61) to test the associations between *L. iners*, *G. vaginalis*, Shannon diversity index, and CST with SNPs using linear regression, and association between L. crispatus and SNPs using logistic regression, adjusting for age and principal components. We assumed an additive genetic effect model for all analyses. To account for genotype uncertainty, the allelic dosages from imputed SNPs were analyzed using Mach2QTL for linear regression and Mach2DAT for logistic regression (see “Web resources” below). SNPs with a *P* value of less than 1.49 × 10^−7^ (0.05/336,151) were declared genome-wide significant. The software R (3.5.0) (62) and LocusZoom (63) (hg19/1000 Genomes AFR) were used for graphing. We calculated the count of SNPs meeting a range of *P* value thresholds (see Table S5 in the supplemental material). Additionally, we estimated the minimum sample sizes needed to reach Bonferroni level significance (1.49 × 10^−7^) based on linear regression of each trait and SNPs at 80%, 90%, and 95% power thresholds (Table S6). We were unable to generate a reliable sample size estimate for L. crispatus due to the odds ratio estimate and low prevalence of the trait.

### Gene, pathway, and disease/phenotype analyses.

We performed gene, pathway, and disease/phenotype analyses using SKAT-O (64) and WebGestalt (65) to examine the combined genetic effects on the underlying biological mechanisms influencing the vaginal microbiome traits. We mapped all directly genotyped SNPs to autosomal genes based on the genomic positions of the GRCh37/hg19 assembly and included a ±20-kb gene boundary to capture proximal regulatory and functional elements that contribute to gene regulation, resulting in 21,213 genes for analysis. Gene set associations were performed using SKAT-O, adjusting for age and the first three principal components. Overrepresentation analyses of pathways (Kyoto Encyclopedia of Genes and Genomes [KEGG] and Reactome) and diseases/phenotypes (DisGeNET, GLAD4U, Online Mendelian Inheritance in Man [OMIM], and Human Phenotype Ontology) were analyzed using WebGestalt. Genes with a *P* value of less than 2.36 × 10^−6^ (0.05/21,213) were declared significant. Pathways, diseases, and phenotypes with a false-discovery rate (FDR) *P* value of <0.05 after Benjamini-Hochberg multiple testing adjustment were declared significant. The top five genes, pathways, and diseases/phenotypes from SKAT-O and WebGestalt are reported.

### Web resources.

The Web resources are as follows: Mach2DAT (http://csg.sph.umich.edu/abecasis/MACH/download/) and Mach2QTL (http://csg.sph.umich.edu/abecasis/MACH/download/).

### Data availability.

Raw sequence data files are available in the Sequence Read Archive (National Center for Biotechnology Information; BioProject identifier PRJNA516684).

10.1128/mSystems.00502-20.9 DATA SET S1 Final analytic data set GWAS VMB (*n* = 171). Note that this is an Excel file. Download Data Set S1, XLS file, 0.05 MB. Copyright © 2020 Mehta et al. 2020 Mehta et al. This content is distributed under the terms of the Creative Commons Attribution 4.0 International license.
