# Supplementary figures and images for: Host Genetic Factors Associated with Vaginal Microbiome Composition in Kenyan Women
Source: mSystems. 2020 Jul 28;5(4):e00502-20. doi: 10.1128/mSystems.00502-20 (PMC7394359; doi:10.1128/mSystems.00502-20)

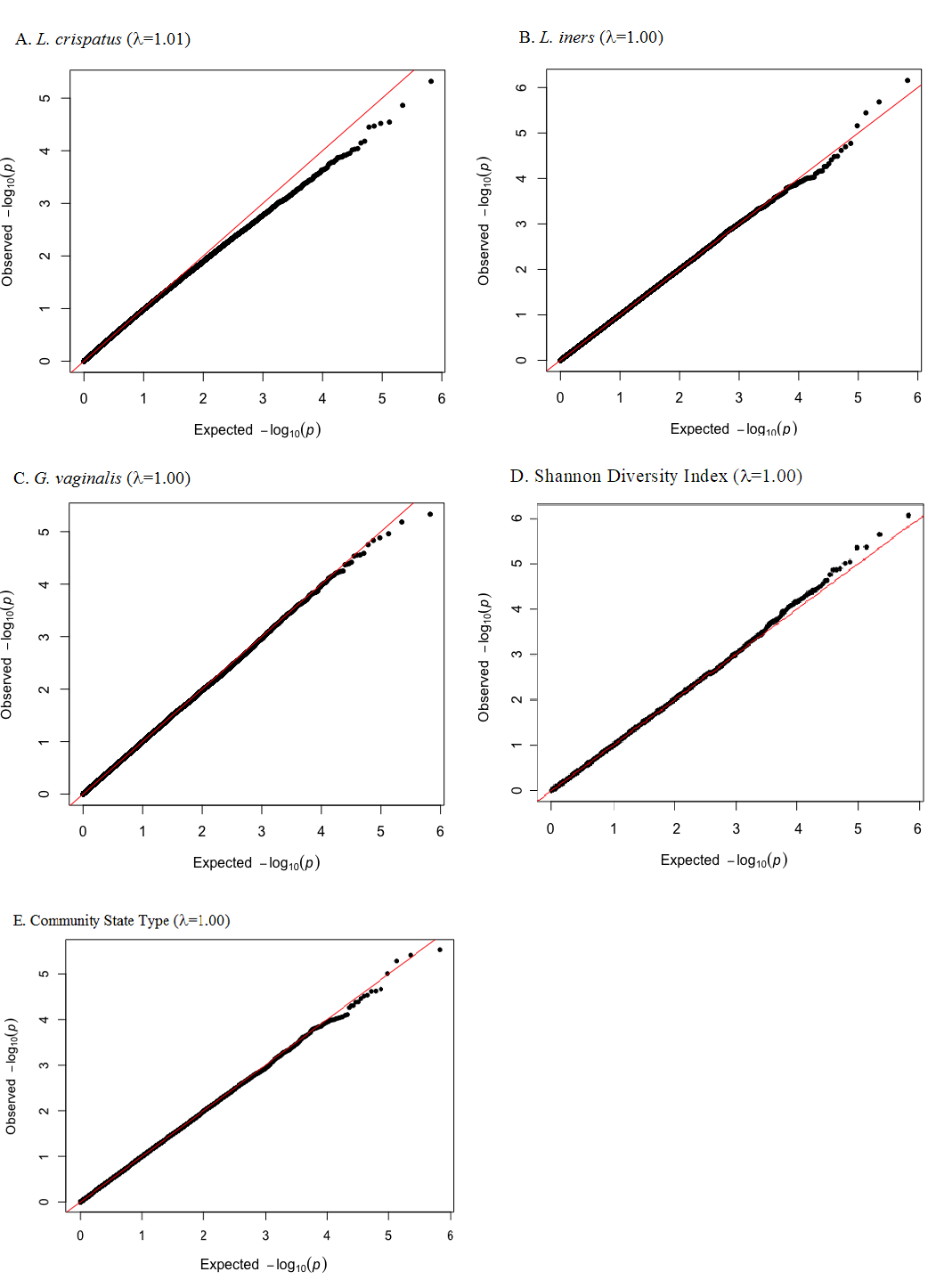

Supplement: FIG S1 [file mSystems.00502-20-sf001.tif]

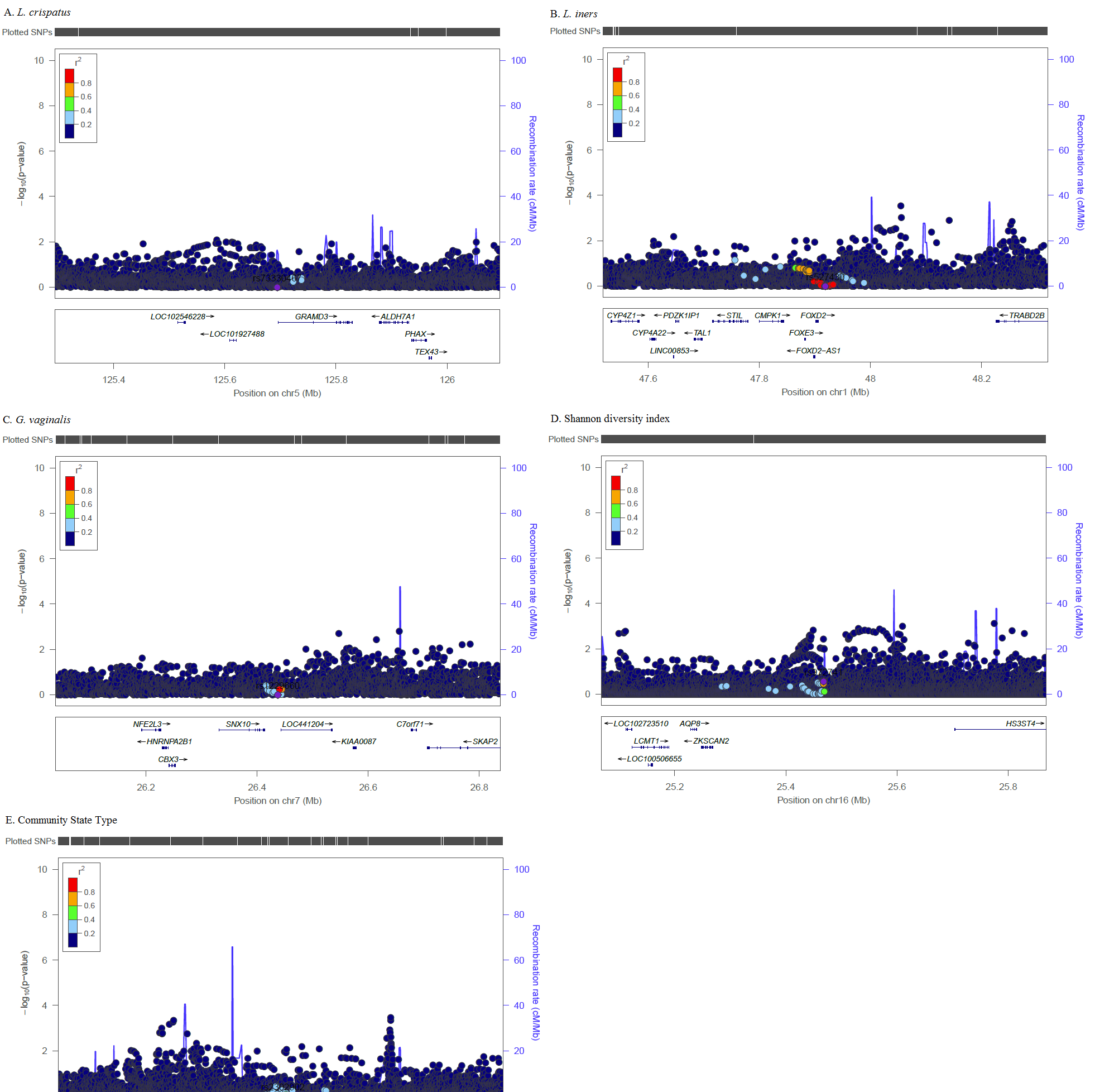

Supplement: FIG S2 [file mSystems.00502-20-sf002.tif]
