# Supplementary material for: Host Genetic Factors Associated with Vaginal Microbiome Composition in Kenyan Women
Source: mSystems. 2020 Jul 28;5(4):e00502-20. doi: 10.1128/mSystems.00502-20 (PMC7394359; doi:10.1128/mSystems.00502-20)
Supplement: TABLE S1 [file mSystems.00502-20-st001.docx]

**Supplemental Table 1.** **Sensitivity analysis of the top GWAS SNPs adjusting for HIV and HSV-2 status.**

|  | SNP | Chr | Position | A1/A2 | Age, PC1, PC2, PC3 | | Age, PC1, PC2, PC3, HIV, HSV | |
| --- | --- | --- | --- | --- | --- | --- | --- | --- |
|  |  |  |  |  | β | *P* | β | *P* |
| *L. crispatus* |  |  |  |  |  |  |  |  |
|  | rs73330467 | 5 | 125694420 | G/T | 11.58*^a^* | 4.79E-06 | 10.14*^a^* | 2.09E-05 |
| *L. iners* |  |  |  |  |  |  |  |  |
|  | rs527430 | 1 | 47918821 | A/G | 1.05 | 6.98E-07 | 1.03 | 2.20E-06 |
|  | rs77007265 | 2 | 31384829 | C/A | -0.93 | 2.07E-06 | -0.92 | 5.25E-06 |
|  | rs17010778 | 2 | 31384974 | C/T | -1.00 | 3.60E-06 | -0.99 | 8.08E-06 |
|  | rs12221275 | 10 | 122972398 | A/C | 2.22 | 6.95E-06 | 2.21 | 8.29E-06 |
| *G. vaginalis* |  |  |  |  |  |  |  |  |
|  | rs1229660 | 7 | 26437429 | C/T | -0.99 | 4.65E-06 | -0.91 | 1.45E-05 |
|  | rs10414170 | 19 | 57246309 | C/A | -0.62 | 6.56E-06 | -0.67 | 4.56E-07 |
| Shannon Diversity Index |  |  |  |  |  |  |  |  |
|  | rs7632135 | 3 | 154455745 | G/A | -0.59 | 4.37E-06 | -0.55 | 2.08E-05 |
|  | rs3097137 | 5 | 73330562 | A/G | 0.56 | 4.25E-06 | 0.53 | 9.52E-06 |
|  | rs112627544 | 7 | 1929410 | T/G | -0.47 | 9.04E-06 | -0.43 | 3.54E-05 |
|  | rs6970796 | 7 | 1947895 | T/C | -0.50 | 2.25E-06 | -0.46 | 1.72E-05 |
|  | rs56952063 | 14 | 95107145 | C/T | -0.90 | 9.65E-06 | -0.81 | 8.58E-05 |
|  | rs972741 | 16 | 25468083 | A/G | 0.66 | 8.52E-07 | 0.67 | 3.74E-07 |
| Community State Type |  |  |  |  |  |  |  |  |
|  | rs419816 | 5 | 52571758 | C/T | 0.45 | 9.99E-06 | 0.43 | 1.19E-05 |
|  | rs1929353 | 9 | 3759975 | G/A | 0.35 | 9.51E-06 | 0.33 | 1.97E-05 |
|  | rs2302902 | 12 | 96617304 | T/C | 0.41 | 3.09E-06 | 0.35 | 4.56E-05 |
