# Supplementary material for: Host Genetic Factors Associated with Vaginal Microbiome Composition in Kenyan Women
Source: mSystems. 2020 Jul 28;5(4):e00502-20. doi: 10.1128/mSystems.00502-20 (PMC7394359; doi:10.1128/mSystems.00502-20)
Supplement: TABLE S2 [file mSystems.00502-20-st002.docx]

**Supplemental Table 2. Comparison of top SNPs across vaginal microbiome traits**

| Original Trait | SNP | Chr | Position | *L. crispatus* | | *L. iners* | | *G. vaginalis* | | Shannon | | CST | |
| --- | --- | --- | --- | --- | --- | --- | --- | --- | --- | --- | --- | --- | --- |
|  |  |  |  | OR | *P* | β | *P* | β | *P* | β | *P* | β | *P* |
| *L. crispatus* | rs73330467 | 5 | 125694420 | 11.58 | *4.79E-06* | 0.00 | 9.83E-01 | -0.72 | **3.06E-03** | -0.49 | 1.93E-02 | 0.55 | **5.35E-05** |
| *L. iners* | rs527430 | 1 | 47918821 | 0.86 | 7.80E-01 | 1.05 | *6.98E-07* | -0.55 | 3.02E-02 | -0.79 | **1.95E-04** | 0.30 | 3.29E-02 |
| *L. iners* | rs77007265 | 2 | 31384829 | 1.00 | 9.95E-01 | -0.93 | *2.07E-06* | 0.18 | 4.54E-01 | 0.68 | **6.38E-04** | -0.28 | 3.54E-02 |
| *L. iners* | rs17010778 | 2 | 31384974 | 0.80 | 6.94E-01 | -1.00 | *3.60E-06* | 0.19 | 4.60E-01 | 0.62 | 4.64E-03 | -0.38 | 8.73E-03 |
| *L. iners* | rs12221275 | 10 | 122972398 | 0.80 | 8.53E-01 | 2.22 | *6.95E-06* | -1.05 | 7.33E-02 | -2.14 | **1.26E-05** | 0.45 | 1.76E-01 |
| *G. vaginalis* | rs1229660 | 7 | 26437429 | 1.61 | 2.82E-01 | 0.42 | 2.63E-02 | -0.99 | *4.65E-06* | -0.53 | 4.77E-03 | 0.20 | 1.02E-01 |
| *G. vaginalis* | rs10414170 | 19 | 57246309 | 1.02 | 9.53E-01 | 0.30 | 1.17E-02 | -0.62 | *6.56E-06* | -0.28 | 1.81E-02 | 0.14 | 8.08E-02 |
| Shannon | rs7632135 | 3 | 154455745 | 0.99 | 9.85E-01 | 0.45 | **7.12E-04** | -0.49 | **1.23E-03** | -0.59 | *4.37E-06* | 0.20 | 2.67E-02 |
| Shannon | rs3097137 | 5 | 73330562 | 1.04 | 8.87E-01 | -0.44 | **4.77E-04** | 0.41 | 4.98E-03 | 0.56 | *4.25E-06* | -0.34 | **3.43E-05** |
| Shannon | rs112627544 | 7 | 1929410 | 1.32 | 2.92E-01 | 0.23 | 3.66E-02 | -0.37 | **3.12E-03** | -0.47 | *9.04E-06* | 0.21 | **2.98E-03** |
| Shannon | rs6970796 | 7 | 1947895 | 1.02 | 9.46E-01 | 0.21 | 5.32E-02 | -0.38 | **2.42E-03** | -0.50 | *2.25E-06* | 0.22 | **1.98E-03** |
| Shannon | rs56952063 | 14 | 95107145 | 0.60 | 3.51E-01 | 0.80 | **1.10E-04** | -0.70 | 3.87E-03 | -0.90 | *9.65E-06* | 0.43 | **1.69E-03** |
| Shannon | rs972741 | 16 | 25468083 | 1.17 | 6.40E-01 | -0.47 | **6.40E-04** | 0.25 | 1.16E-01 | 0.66 | *8.52E-07* | -0.13 | 1.67E-01 |
| CST | rs419816 | 5 | 52571758 | 2.38 | 1.45E-02 | 0.03 | 8.72E-01 | -0.38 | 3.84E-02 | -0.30 | 5.24E-02 | 0.45 | *9.99E-06* |
| CST | rs1929353 | 9 | 3759975 | 3.05 | **2.09E-04** | 0.09 | 4.42E-01 | -0.18 | 2.00E-01 | -0.21 | 7.85E-02 | 0.35 | *9.51E-06* |
| CST | rs2302902 | 12 | 96617304 | 2.10 | 1.84E-02 | 0.00 | 9.99E-01 | -0.34 | 3.41E-02 | -0.23 | 9.21E-02 | 0.41 | *3.09E-06* |
