# Supplementary material for: Host Genetic Factors Associated with Vaginal Microbiome Composition in Kenyan Women
Source: mSystems. 2020 Jul 28;5(4):e00502-20. doi: 10.1128/mSystems.00502-20 (PMC7394359; doi:10.1128/mSystems.00502-20)
Supplement: TABLE S4 [file mSystems.00502-20-st004.docx]

**Supplemental Table 4. Top genes from gene level analysis for each vaginal microbiome trait.**

| Trait | Gene | *P* |
| --- | --- | --- |
| *L. crispatus* |  |  |
|  | *PARM1* | 1.41E-05 |
|  | *ITLN2* | 2.04E-05 |
|  | *LOC101928372* | 2.04E-05 |
|  | *LINC00923* | 2.67E-05 |
|  | *ANKRD33* | 4.13E-05 |
| *L. iners* |  |  |
|  | *LRRC49* | 3.79E-05 |
|  | *RRP12* | 2.26E-04 |
|  | *CTD-2297D10.2* | 2.58E-04 |
|  | *MT4* | 2.73E-04 |
|  | *ARL5B* | 3.13E-04 |
| *G. vaginalis* |  |  |
|  | *GPR137* | 1.78E-05 |
|  | *KCNK4* | 1.78E-05 |
|  | *TEX40* | 1.78E-05 |
|  | *THRIL* | 2.03E-04 |
|  | *BRI3BP* | 2.04E-04 |
| Shannon Diversity Index | | |
|  | *TAF3* | 1.24E-04 |
|  | *HNRNPA3* | 2.34E-04 |
|  | *LOC101927040* | 5.63E-04 |
|  | *HEY1* | 7.10E-04 |
|  | *TEFM* | 7.18E-04 |
| Community State Type | | |
|  | *LINC01098* | 6.53E-05 |
|  | *LINC01099* | 7.10E-05 |
|  | *HAND2* | 2.71E-04 |
|  | *COMMD10* | 5.36E-04 |
|  | *ZNF138* | 5.36E-04 |
