# Supplementary material for: Host Genetic Factors Associated with Vaginal Microbiome Composition in Kenyan Women
Source: mSystems. 2020 Jul 28;5(4):e00502-20. doi: 10.1128/mSystems.00502-20 (PMC7394359; doi:10.1128/mSystems.00502-20)
Supplement: TABLE S5 [file mSystems.00502-20-st005.docx]

**Supplemental Table 5. Count of top single nucleotide polymorphisms (SNPs) by *P*-value threshold for each vaginal microbiome trait.**

| *P* ^*^ | *L. crispatus* | *L. iners* | *G. vaginalis* | Shannon Diversity Index | Community State Type |
| --- | --- | --- | --- | --- | --- |
| 5.00x10^-8^ | 0 | 0 | 0 | 0 | 0 |
| 1.49x10^-7^ | 0 | 0 | 0 | 0 | 0 |
| 1.00x10^-6^ | 0 | 1 | 0 | 1 | 0 |
| 1.00x10^-5^ | 1 | 4 | 2 | 6 | 3 |
| 1.00x10^-4^ | 11 | 24 | 32 | 50 | 24 |
| 1.00x10^-3^ | 186 | 344 | 310 | 346 | 322 |
