# Supplementary material for: Host Genetic Factors Associated with Vaginal Microbiome Composition in Kenyan Women
Source: mSystems. 2020 Jul 28;5(4):e00502-20. doi: 10.1128/mSystems.00502-20 (PMC7394359; doi:10.1128/mSystems.00502-20)
Supplement: TABLE S6 [file mSystems.00502-20-st006.docx]

**Supplemental Table 6. Power table representing the minimum sample sizes needed to reach Bonferroni significance^*^ for each vaginal microbiome trait, expressed at three statistical power settings: 80%, 90%, and 95% power.**

| Trait | SNP | Chr | Position | Gene | β | *P* | Minimum sample size to achieve specified power | | | N Extra at 95% Power^**^ |
| --- | --- | --- | --- | --- | --- | --- | --- | --- | --- | --- |
|  |  |  |  |  |  |  | 80% | 90% | **95%** |  |
| *L. iners* |  |  |  |  |  |  |  |  |  |  |
|  | rs527430 | 1 | 47918821 | *FOXD2-TRABD2B* | 1.05 | 6.98x10^-7^ | 224 | 255 | 283 | 112 |
|  | rs77007265 | 2 | 31384829 | *GALNT14-CAPN14* | -0.93 | 2.07x10^-6^ | 261 | 298 | 330 | 159 |
|  | rs17010778 | 2 | 31384974 | *GALNT14-CAPN14* | -1.00 | 3.60x10^-6^ | 268 | 305 | 339 | 168 |
|  | rs12221275 | 10 | 122972398 | *WDR11-FGFR2* | 2.22 | 6.95x10^-6^ | 286 | 326 | 361 | 190 |
| *G. vaginalis* |  |  |  |  |  |  |  |  |  |  |
|  | rs1229660 | 7 | 26437429 | *SNX10-LOC441204* | -0.99 | 4.65x10^-6^ | 281 | 321 | 356 | 185 |
|  | rs10414170 | 19 | 57246309 | *ZNF835-ZIM2-AS1* | -0.62 | 6.56x10^-6^ | 286 | 326 | 361 | 190 |
| Shannon Diversity Index | |  |  |  |  |  |  |  |  |  |
|  | rs7632135 | 3 | 154455745 | *GPR149-MME* | -0.59 | 4.37x10^-6^ | 171 | 193 | 213 | 42 |
|  | rs3097137 | 5 | 73330562 | *ARHGEF28-LINC01335* | 0.56 | 4.25x10^-6^ | 176 | 199 | 220 | 49 |
|  | rs112627544 | 7 | 1929410 | *MAD1L1* | -0.47 | 9.04x10^-6^ | 183 | 208 | 230 | 59 |
|  | rs6970796 | 7 | 1947895 | *MAD1L1* | -0.50 | 2.25x10^-6^ | 171 | 184 | 203 | 32 |
|  | rs56952063 | 14 | 95107145 | *SERPINA13P* | -0.90 | 9.65x10^-6^ | 191 | 217 | 240 | 69 |
|  | rs972741 | 16 | 25468083 | *ZKSCAN2-HS3ST4* | 0.66 | 8.52x10^-7^ | 171 | 171 | 188 | 17 |
| Community State Type | |  |  |  |  |  |  |  |  |  |
|  | rs419816 | 5 | 52571758 | *LOC257396-FST* | 0.45 | 9.99x10^-6^ | 297 | 339 | 376 | 205 |
|  | rs1929353 | 9 | 3759975 | *RFX3-AS1-GLIS3* | 0.35 | 9.51x10^-6^ | 297 | 338 | 375 | 204 |
|  | rs2302902 | 12 | 96617304 | *ELK3* | 0.41 | 3.09x10^-6^ | 262 | 299 | 331 | 160 |
